# Fingolimod Exerts Therapeutic Effects on Autistic Mice via Improving the Structure and Function of Meningeal Lymphatics

**DOI:** 10.1111/cns.70649

**Published:** 2025-11-11

**Authors:** Chen Hong, Han‐Lian Xiao, Zhi‐Hui Sun, Ruo‐Bing Guo, Xi‐Yue Zhang, Juan Ji, Xiu‐Lan Sun

**Affiliations:** ^1^ Jiangsu Key Laboratory of Neurodegeneration Nanjing Medical University Nanjing Jiangsu China; ^2^ Department of Pharmacy General Hospital of Ningxia Medical University Yinchuan Ningxia China; ^3^ Nanjing University of Chinese Medicine, The Affiliated Hospital of Nanjing University of Chinese Medicine Nanjing Jiangsu China

**Keywords:** autism spectrum disorder, fingolimod, maternal immune activation, meningeal lymphatic vessels, ventricular enlargement

## Abstract

**Background:**

Accumulating evidence suggests a correlation between maternal infection during pregnancy and an increased risk of autism spectrum disorder (ASD) in offspring. Our previous studies have demonstrated that maternal immune activation (MIA) induces autism‐like behaviors in offspring mice, accompanied by significant lateral ventricular enlargement and cerebrospinal fluid (CSF) circulation deficits. As a critical pathway for CSF drainage, the role of meningeal lymphatic vessels in the pathophysiology of ASD remains uncharacterized. Fingolimod (FTY720), a clinically used immunomodulator, has been shown to ameliorate autism‐like behaviors, but its underlying mechanism remains unclear.

**Methods:**

We utilized an MIA‐induced autism‐like offspring mouse model. Autism‐like behaviors in the mice were assessed using three‐chamber social interaction, marble burying, grooming and other related behavioral tests. The size of the lateral ventricles was evaluated by magnetic resonance imaging and hematoxylin and eosin staining. The structure and function of meningeal lymphatic vessels were examined using immunofluorescence and in vivo visible light imaging. Lymphangiogenesis was investigated through techniques such as Western blotting, tube formation assays, sprouting experiments and other relevant methods.

**Results:**

FTY720 not only alleviates autism‐like behaviors and lateral ventricular dilation, but also significantly restores the structural integrity and drainage function of meningeal lymphatic vessels in MIA offspring. Furthermore, in vitro experiments reveal that FTY720 promotes lymphangiogenesis by targeting the S1PR3 receptor to inhibit the expression of thrombospondin‐1 (TSP1), a lymphangiogenesis‐related inhibitor.

**Conclusion:**

FTY720 acts on the S1PR3 receptor to inhibit TSP1, thereby improving the structure and function of meningeal lymphatic vessels and alleviating autism‐like behaviors and lateral ventricular dilation.

## Graphical abstract

FTY720 binds to the S1PR3 receptor, inhibits the levels of the lymphangiogenesis‐inhibiting factor tsp1, and promotes the sprouting and tube formation of lymphatic endothelial cells. This action improves the structure and function of meningeal lymphatic vessels, thereby alleviating social deficiencies, anxiety, repetition of stereotyped behaviors, and ventricular enlargement in ASD mice.

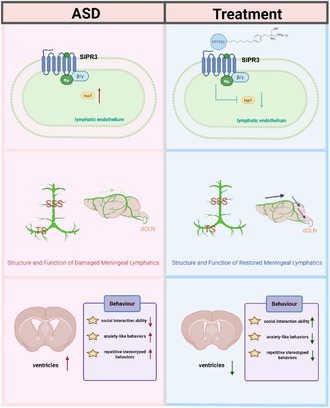

## 1 Introduction

Autism spectrum disorder (ASD) is a neurodevelopmental disorder with onset during infancy and early childhood. Patients with ASD typically exhibit deficits in social communication, restricted interests or activities, and repetitive and stereotyped behaviors, with severity and accompanying abilities distributed along a spectrum [1, 2]. Recent clinical and animal studies have both indicated that ventriculomegaly is associated with ASD: children with a prenatal diagnosis of isolated fetal ventricular enlargement are more likely to exhibit ASD phenotypes [3]; Ventriculomegaly has been observed in children with ASD [4] and in Katnal2‐knockout mouse models of autism [5, 6]. Our previous work also revealed that the ASD model mice induced by maternal immune activation (MIA) exhibited persistent ventricular enlargement from birth to adulthood, which is closely associated with dysfunction of the choroid plexus and ependymal [7]. However, whether additional intracerebral structures contribute to this process remains to be determined.

Similarly, recent studies have shown that children with ASD also exhibit glymphatic dysfunction [8, 9], a dysfunction linked to disease progression and ventricular enlargement across multiple neurological conditions [10, 11]. Yet the glymphatic system itself lacks authentic lymphatic vessel architecture. In 2015, the laboratories of Kari Alitalo [12] and Jonathan Kipnis [13] independently identified an extensive network of meningeal lymphatic vessels (mLVs) that drain cerebrospinal fluid together with immune cells and macromolecules to the deep cervical lymph nodes, thereby establishing a critical highway for central nervous system–peripheral immune and metabolic exchange. Subsequently, in Parkinson's disease models [14] burdened with α‐synuclein and in Alzheimer's disease models [15] laden with Aβ and hyper‐phosphorylated tau, mLVs were found to be structurally compromised and functionally impaired; restoring their integrity markedly reduced pathological protein loads and ameliorated disease phenotypes. Given that the ASD brain is characterized by chronic low‐grade neuroinflammation [16, 17] and accumulation of immune cells [18], mLVs (serving as the principal exit route for cerebral immune and macromolecular waste) are highly likely to be structurally or functionally deficient in ASD, although direct evidence from ASD models is still lacking.

Fingolimod (FTY720) is an immunomodulatory drug for the treatment of relapsing forms of multiple sclerosis (MS) [19]. Its pharmacological effects are primarily mediated through the modulation of sphingosine‐1‐phosphate (S1P) receptors (S1PRs). Studies have shown that FTY720 holds potential therapeutic value in the treatment of central nervous system disorders, including ischemic stroke [20] and Parkinson's disease [21]. Recent research has also demonstrated that FTY720, also can significantly ameliorate behavioral abnormalities and pathological features in animal models of ASD [22, 23]. However, the exact mechanisms of action remain to be fully elucidated. Notably, S1PRs play a key role in regulating the expression of lymphangiogenesis‐related factors [24, 25, 26, 27, 28], implying that FTY720 may modulate the formation of meningeal lymphatic vessels. Therefore, this study focuses on exploring whether FTY720 can improve ASD‐like behavioral phenotypes and modulate the meningeal lymphatic system in maternal immune activation (MIA)‐induced ASD offspring, as well as elucidating the underlying molecular mechanisms.

## 2 Materials and Methods

### 2.1 Primers (Tsingke Biotechnology Co., Beijing, China)

**Table:** 

Gene	Sequence
s1pr1	F: ATGGTGTCCACTAGCATCCC
	R: CGATGTTCAACTTGCCTGTGTAG
s1pr2	F: CCATCGCCATCGAGAGACAAG
	R: CACGTAGTGCTTAGCATAGAGAG
s1pr3	F: ACTCTCCGGGAACATTACGAT
	R: CAAGACGATGAAGCTACAGGTG
s1pr4	F: CCCTGGCCGTGTTCAACTC
	R: ACCGAGAAGTCCGAAAACTGT
s1pr5	F: GCTTTGGTTTGCGCGTGAG
	R: GGCGTCCTAAGCAGTTCCAG
ang1	F: CACATAGGGTGCAGCAACCA
	R: CGTCGTGTTCTGGAAGAATGA
igf1	F: CTGGACCAGAGACCCTTTGC
	R: GGACGGGGACTTCTGAGTCTT
pdgf2	F: CATCCGCTCCTTTGATGATCTT
	R: GTGCTCGGGTCATGTTCAAGT
vegfc	F: GAGGTCAAGGCTTTTGAAGGC
	R: CTGTCCTGGTATTGAGGGTGG
tsp1	F: GTTCGTCGGAAGGATTGTTA
	R: TCTATTCCAATGGCAACGAG
tsp2	F: CAGAGTACTGGCGTCGGTCA
	R: ATAAGATCGCAGCCCACATACAG
gapdh	F: TGTGTCCGTCGTGGATCTGA
	R: CCTGCTTCACCACCTTCTTGAT
β‐Actin	F: GGCTGTATTCCCCTCCATCG
	R: CCAGTTGGTAACAATGCCATGT

### 2.2 Antibody

Anti‐PSD95 antibody (1:1000, Abcam, ab12093, Cambridge, United Kingdom); Anti‐β‐tubulin antibody (1:1000, Proteintech, 10094‐1‐AP, Chicago, USA); Anti‐LYVE1 antibody (1:500, Cell Signaling Technology, 67538S, Danvers, USA); Rabbit secondary antibody (1:5000, Proteintech, SA00001‐2, Chicago, USA); Anti‐PROX1 antibody (1:500, Proteintech, 11067‐2‐AP, Chicago, USA); Anti‐β‐actin antibody (1:1000, Proteintech, 20536‐1‐AP, Chicago, USA); Fluorescent Rabbit secondary antibody (1:500, Thermo Fisher Scientific, A‐21206, Massachusetts, USA).

### 2.3 Experimental Animals and Animal Model

The SPF‐grade C57BL/6 mice used in this study were obtained from the Experimental Animal Center of Nanjing Medical University and maintained under standard barrier conditions (20°C–26°C, 30%–70% humidity, 12 h light/dark cycle). All procedures were approved by the Institutional Animal Care and Use Committee of Nanjing Medical University (IACUC2004037‐1) and conducted in accordance with the Guide for the Care and Use of Laboratory Animals.

In this study, 7‐ to 8‐week‐old male and female C57BL/6 mice were co‐housed at a ratio of 1:2 for breeding. On gestational day 12.5 (E12.5), pregnant dams were intraperitoneally injected with either PBS (control group, Phosphate Buffered Saline, G0002‐15, Servicebio) or Poly I:C (0.02 mg/g, MIA model group, P9582, Sigma, Vehicle: PBS). After natural delivery, the offspring from the PBS control group and the MIA model group were obtained. For male offspring in the MIA group, a subset of individuals was randomly selected at postnatal Week 3 for FTY720 intervention treatment (dosing regimen: intraperitoneal injection at 1 mg/kg [22, 23], three times per week for four consecutive weeks, Vehicle: 0.9% NaCl + 0.2% DMSO, S5002, Selleck Chemicals).

### 2.4 Treatment of SVEC4‐10 Cells

The SVEC4‐10 cells used in this study were obtained from the China Center for Type Culture Collection (RRID number: CVCL_4393, CCRID number: 4201MOU‐CCTCC00212). The cells were divided into three treatment groups: (1) the control group (untreated control); (2) the IFN‐γ group (stimulated with 100 ng/mL IFN‐γ for 24 h to simulate the in vivo high IFN‐γ environment, 315‐05, Pepro Tech); and (3) the IFN‐γ + FTY720 group (treated with 100 nM FTY720 in combination with 100 ng/mL IFN‐γ for 24 h). To investigate the receptor mechanisms, the S1PR1 antagonist W146 TFA (GC45155, GLPBIO) or the S1PR3 antagonist CAY10444 (GC18757, GLPBIO) was pre‐incubated for 2 h before treatment with IFN‐γ and FTY720.

### 2.5 Three‐Chamber Social Test

The three‐chamber social test was employed to assess the social interaction and social novelty of mice [29], and the experiment was divided into two stages. Stage 1 (Social Interaction Test): The experimental mouse was first placed in the central chamber of the three‐chamber social apparatus, with all partition doors open, allowing it to freely explore the entire apparatus for 5–10 min to acclimate to the environment. Subsequently, the mouse was returned to the central chamber and the side chamber doors were closed. A novel mouse (mouse 1) was randomly placed into the cage in one of the side chambers. After the partition doors were reopened, the experimental mouse was allowed to freely explore for 10 min. During this period, the time spent sniffing each cage was recorded using the ANY‐maze video tracking system (Stoelting, USA). Social interaction was assessed by calculating the percentage of time the test mouse spent sniffing the cage containing mouse 1 relative to the total time spent sniffing all cages. Stage 2 (Social Novelty Test): The experimental mouse was again returned to the central chamber with the side chamber doors closed. The apparatus was thoroughly cleaned with alcohol and paper towels to eliminate any residual odors. A new novel mouse (mouse 2) was then placed into the cage in the other side chamber. After the partition doors were reopened, the mouse was allowed to freely explore for 10 min, with sniffing times recorded using the ANY‐maze system. Social novelty was represented by the percentage of time the test mouse spent sniffing the cage containing mouse 2 relative to the total time spent sniffing all cages, which effectively reflects the mouse's preference for social novelty.

### 2.6 Open Field Test

To assess the locomotor ability and exploratory behavior of the mice, the animals were transferred to the testing room 3 days prior to the experiment and were gently handled to acclimate them to the environment and minimize stress responses. For the formal test, each mouse was placed in the central area of a blue opaque open‐field chamber (50 × 50 × 60 cm). The locomotor activity was immediately recorded for 15 min using the TopScan Realtime Option Version 2.00 behavioral analysis system (Clever Sys, USA) equipped with a video camera [29]. Behavioral assessments were conducted by analyzing the total distance traveled by the mice (reflecting locomotor ability) and the total time spent in the central area of the chamber (reflecting exploratory behavior and anxiety‐like behavior).

### 2.7 Elevated Plus‐Maze Test

The elevated plus maze (EPM) test was employed to assess anxiety‐like behavior in mice. The maze consisted of two open arms (35 × 5 × 15 cm) and two closed arms (of the same dimensions), all constructed from white plastic material, with the central platform elevated 60 cm above the ground. During the experiment, each mouse was placed on the central platform facing an open arm and allowed to freely explore for 10 min. The locomotor activity of the mice was recorded in real time using the Noldus EthoVision XT behavioral analysis system (Noldus, Netherlands) [30]. The distance traveled into the open arms was quantified as a proportion of the total distance traveled into all four arms (distance traveled into open arms/total distance traveled into all arms), and this ratio was used as a quantitative measure of anxiety‐like behavior.

### 2.8 Marble Burying Test

The experiment utilized transparent cages (36 × 20 × 13 cm) with a 5‐cm layer of bedding material. Twenty transparent glass marbles (15 mm in diameter) were evenly arranged on the bedding surface in a 4 × 5 matrix. Each subject mouse was placed individually into the cage and allowed to move freely for 30 min before being removed. The marble burying index was then calculated (scoring criteria: a marble was assigned 1 point if it was buried to a depth of ≥ 2/3 by the bedding material; otherwise it was assigned 0 points) [31].

### 2.9 Self‐Grooming Test

The self‐grooming test was employed to assess repetitive stereotyped behaviors in mice. Each subject mouse was placed individually in a cage with clean, fresh bedding material. After a 5‐min acclimation period, the spontaneous behavior of the mice was continuously recorded for 10 min using the Noldus EthoVision XT behavioral analysis system (Noldus, Netherlands) [32]. The total duration spent by the mice in scratching their face, head, or other body parts was quantified through video analysis, thereby providing a measure of the intensity and frequency of self‐grooming behavior.

### 2.10 Mouse Ultrasonic Vocalization Detection Test

Ultrasonic vocalization detection test was used to record and analyze Ultrasonic Vocalizations (USVs) produced by mice. USVs were detected on postnatal Days 3, 5, 7, 9, and 11. The pups were removed from their mothers and left alone for 30 min before the experiment began. The experiment was officially started; the mouse ultrasonic detection instrument was connected, the mouse was put into the iron box with a sponge sound insulation layer on the inner wall, the ultrasonic receiver was installed, and the ultrasonic recording software Avisoft USV Recorder was started to record [33]. After the experiment, it was saved as a *.wav sound file, and Deep Squeak was used to extract the ultrasonic sound characteristics of the mice. This feature extraction was carried out based on each independent ultrasonic sound produced by the mice. The Time of Duration, Mean Power and the difference between the highest and lowest frequency of vocalization (ΔFrequency) were recorded. At the same time, three spectral indices, including Slope, Sinuosity and Tonality, were introduced to quantitatively describe the waveform changes in the phonation spectrogram.

### 2.11 Reciprocal Social Interaction Test

Reciprocal social interaction test was used to evaluate the reciprocal behavior of animals or humans in social interactions. The test mice were individually placed in a clean transparent plastic cage for 10 min to acclimate. Then, an unfamiliar same‐sex mouse of similar age and weight was introduced into the cage, which was called the stimulus mouse. The test mice and the stimulus mice interacted freely for 10 min. The behavior of mice was recorded by Noldus Ethovision XT software, and the movement trajectory was statistically analyzed [34]. The duration of social interactions—including nose‐to‐nose sniffing, following, chasing, mounting, grooming, and climbing over/under—between the test mouse and the stimulus mouse was used as the index of social competence.

### 2.12 Magnetic Resonance Imaging

A 7T small‐animal magnetic resonance imaging (MRI) system (BioSpec 7T/20 USR, Bruker, Germany) was employed to perform structural brain scans in mice. During the experiment, anesthesia was maintained with 5% isoflurane (R510‐22‐10, RWD Life Science), and the mice were positioned in a prone position and secured on the scanning bed with their heads aligned. T2‐weighted imaging sequences were used to acquire brain structural images, with the following scanning parameters: repetition time (TR) 2500 ms, echo time (TE) 35 ms, flip angle (FA) 90.0°, acquisition time (TA) 4 min, number of excitations (NEX) 3, field of view (FOV) 2 cm, matrix size (MTX) 256, slice thickness 0.8 mm, with no interslice gap for contiguous scanning, and 20–21 consecutive slices were obtained for each mouse. The ventricular regions were delineated and their areas calculated using ImageJ image analysis software. The degree of ventricular enlargement was quantified by measuring and summing the areas of four consecutive slices from the same anatomical locations.

### 2.13 Paraffin Section Staining

The mice were anesthetized, followed by cardiac perfusion with PBS. Subsequently, the brain tissue was extracted and fixed in 4% paraformaldehyde (PFA) for 24 h. After the fixation process was completed, the brain tissue was placed in a white embedding cassette. A series of operations were then performed, including gradient ethanol dehydration for 0.5 h, xylene clarification for 0.5 h, and paraffin infiltration embedding. The fixed brain tissue was then sectioned. When the sections reached the lateral ventricle region, the section thickness was adjusted to 4 μm. The sections were left at room temperature for 2–3 days. After that, the brain tissue sections were first placed in an oven at 67°C for 40 min and then dewaxed in xylene for 15 min, repeated twice. Next, the sections were rehydrated successively with gradient ethanol and distilled water for 3 min each. Subsequently, the sections were stained in hematoxylin (G1076, Servicebio) for 3 min, 1% ethanol hydrochloric acid for 1 s, and eosin (G1076, Servicebio) for 3 min. After each removal from the staining solution, the sections were rinsed with running water to remove excess stain. Finally, the sections were mounted with mounting medium (PBS: glycerol = 1:1) and observed and imaged using an optical microscope (Olympus, Tokyo, Japan).

### 2.14 Immunofluorescence Experiment

The meninges were obtained by anesthetizing the mice, performing cardiac perfusion and fixation as described above, and then extracting the meningeal tissue under a stereomicroscope. For cell acquisition, the treated cells were washed three times with PBS for 5 min each time and then fixed with PFA for 30 min. Both the fixed meninges and cells were washed three times with PBS for 5 min each. Subsequently, they were blocked with 10% donkey serum (diluted in PBS, SL050, Beijing Solarbio Science and Technology Co. Ltd) at room temperature for 1 h. Next, the samples were incubated with anti‐LYVE‐1 primary antibody (1:200) overnight at 4°C. On the following day, after washing with PBS, the corresponding species‐specific fluorescent secondary antibody was applied and incubated in the dark at room temperature for 1 h. After another round of PBS washing, the nuclei were stained with DAPI (ab104139, Abcam) or Hoechst. Finally, the samples were mounted with mounting medium and imaged using a microscope (Zeiss, Germany).

### 2.15 In Vivo Visible Light Imaging of Deep Cervical Lymph Nodes in Mice

Twenty‐four hours before the experiment, the neck region of the mice was depilated. Under anesthesia, the heads of the mice were fixed using a stereotactic apparatus (RWD, Shenzhen, China) and adjusted to a 45°–60° angle with the horizontal plane. The posterior neck region of the mice was then surgically exposed. After making an incision through the skin, the muscle tissue was bluntly dissected to expose the “Y”—shaped structure of the cisterna magna. A glass microneedle loaded with 2.4 μL of OVA—647 fluorescent tracer (O34784, Thermo Fisher Scientific) was used to puncture the dura mater at the intersection of the “Y” shape. The needle was left in place for 2 min before the tracer was infused at a constant rate of 1 μL/min. After the infusion was completed, the needle was left in place for another 2 min before being slowly retracted. The wound was then sealed with biological tissue adhesive. At three time points—15, 30, and 60 min after injection—the fluorescence signal intensity in the cervical lymph node region was quantitatively detected using an in vivo imaging system (IVIS Spectrum, PerkinElmer, USA) with excitation/emission wavelengths set at 640/680 nm.

### 2.16 Fluorescence Imaging of Deep Cervical Lymph Nodes in Mice

The dye injection was performed as described above. Thirty minutes after the injection, the deep cervical lymph nodes were harvested from the mice and then subjected to dehydration in a 20% sucrose solution overnight at 4°C, followed by further treatment in a 30% sucrose solution for 24 h at 4°C. Subsequently, the lymph nodes were continuously sectioned at a thickness of 30 μm using a frozen microtome (Leica, Germany). Fluorescence images of the sections were captured at an excitation wavelength of 647 nm using a confocal laser scanning microscope (Zeiss, Germany). For quantitative analysis, the five sections with the highest fluorescence intensity from each lymph node were selected, and their average fluorescence intensity was calculated to serve as the final measurement value for the fluorescent dye in that lymph node.

### 2.17 Enzyme‐Linked Immunosorbent Assay (ELISA)

The levels of interferons in mouse serum were quantitatively measured using enzyme‐linked immunosorbent assay (ELISA). Whole blood samples were collected by enucleation of the eyeball, allowed to stand at room temperature for 30 min, and then centrifuged at 1000 rpm for 15 min to separate the serum. Following the instructions provided with the commercial ELISA kit (SEA033Mu, SEA222Mu, SEA049Mu, Cloud‐Clone Corp), the concentrations of IFN‐α, IFN‐β, and IFN‐γ in the serum were accurately determined using a multifunctional microplate reader (Thermo Scientific, USA). All samples were assayed in duplicate, and the levels of each interferon were calculated based on a standard curve, with the results expressed in pg/mL.

### 2.18 CCK‐8 Cell Viability Assay

Cell viability of SVEC4‐10 cells was assessed using the CCK‐8 assay. SVEC4‐10 cells were digested, resuspended, and counted, then seeded into a 96‐well plate at a density of 8 × 10^3^ cells per well. After the cells adhered to the plate, they were treated with the S1PR1 antagonist W146 TFA and the S1PR3 antagonist CAY10444 for 24 h. Following the treatment, 10 μL of CCK‐8 reagent (HY‐K0301, MCE) was added to each well, and the plate was incubated at 37°C with 5% CO₂ for 1 h. The absorbance at 450 nm (OD450) was measured using a multifunctional microplate reader (Thermo Fisher Scientific, USA).

### 2.19 Tube Formation Assay

The tube formation ability of lymphatic endothelial cells was evaluated using an in vitro Matrigel‐based assay. The specific procedure was as follows: Sterile pipette tips and 24‐well plates were pre‐chilled at 4°C for 24 h prior to the experiment. On the day of the experiment, Matrigel (354277, BD Biosciences) was thawed on ice for 4 h to ensure complete liquefaction. Subsequently, 200 μL of liquid Matrigel was slowly added to each well of the pre‐chilled 24‐well plate and polymerized at 37°C with 5% CO_2_ for 30 min to form a gel layer. SVEC4‐10 cells were digested with trypsin (cat. no. 9002‐07‐8, Gibco), resuspended, and counted, then seeded onto the Matrigel surface at a density of 1.5 × 10^4^ cells per well and cultured for an additional 2 h. The formation of tubular structures was observed using an inverted microscope (Zeiss, Germany). Seven to eight random fields per well were photographed, and the total length of the tubular structures (μm/field) was measured using ImageJ software for quantitative analysis.

### 2.20 Western Blot Assay (WB)

Western blotting was employed to detect the protein expression levels in SVEC4‐10 cells. The specific procedure was as follows: Cells were lysed on ice for 30 min using pre‐chilled RIPA lysis buffer (containing 1% protease inhibitors, KGP702‐100, Jiangsu KeyGEN Biotech Co. Ltd.), and the lysates were centrifuged at 12,000 *g* for 15 min at 4°C to collect the supernatant. Protein concentrations were determined using the BCA (KGPBCA, Jiangsu KeyGEN Biotech Co. Ltd.) method. Equal amounts of protein samples (30 μg) were separated by 10% SDS‐PAGE gel electrophoresis and then transferred to PVDF membranes (250 mA, 90 min). After transfer, the membranes were blocked with 5% BSA‐TBST (containing 0.1% Tween‐20) blocking solution at room temperature for 1 h, followed by incubation with primary antibodies (diluted 1:500) overnight at 4°C. The next day, the membranes were washed three times with TBST for 15 min each, and then incubated with secondary antibodies (diluted 1:5000) at room temperature for 1 h. After thorough washing, the membranes were developed using ECL chemiluminescent substrate, and the signals were captured using a chemiluminescent imaging system (model). The intensity of the target bands was analyzed using ImageJ software.

### 2.21 Sphere Sprouting Assay

The sprouting capacity of lymphatic endothelial cells was assessed using a three‐dimensional Matrigel spheroid sprouting assay. The preparation of Matrigel and other experimental materials was the same as that described in the tube formation assay. The specific procedure was as follows: SVEC4‐10 cells were digested, counted, and 1.5 × 10^5^ cells were resuspended in 10% FBS (Fetal Bovine Serum, FSS500, ExCell Bio.) DMEM (Dulbecco's Modified Eagle Medium, cat. no. 11960069, Gibco) medium containing 0.24% methylcellulose. A 20 μL droplet of the cell suspension was placed on the inner surface of a culture dish lid (with 5 mL PBS added to the bottom of the dish to maintain humidity) and cultured at 37°C with 5% CO_2_ for 24 h to form spheroids. The spheroids were then collected and mixed with Matrigel (1:4), seeded in the center of the pre‐chilled 24‐well plates, and polymerized at 37°C for 30 min. Subsequently, 500 μL of complete medium containing the respective treatment agents was added to each well, and the cultures were maintained for an additional 48 h. Spheroid sprouting was observed and recorded using an inverted microscope (Nikon, Japan). The sprout length (μm) and the ratio of sprout area to spheroid area were quantitatively analyzed using ImageJ software.

### 2.22 Reverse Transcription Quantitative Polymerase Chain Reaction (RT‐qPCR)

The mRNA expression levels of lymphangiogenesis‐related factors and S1PRs were detected by reverse transcription quantitative polymerase chain reaction (RT‐qPCR). The specific procedure was as follows: Total RNA was extracted from SVEC4‐10 cells subjected to different treatments and meningeal tissues using the Trizol (CW0580S, Kangweicentury Biotechnology Co. Ltd.) method. The concentration and purity of RNA (A260/A280 ratio of 1.8–2.0) were determined using a NanoDrop 2000 spectrophotometer (Thermo Fisher Scientific, USA). The total RNA was reverse‐transcribed into cDNA, and amplification was performed using SYBR Green PCR Master Mix (Kangweicentury, China) on a QuantStudio real‐time PCR system (Thermo Fisher Scientific, USA). The reaction conditions were as follows: initial denaturation at 95°C for 5 min, followed by 40 cycles of 95°C for 15 s and 60°C for 30 s. For lymphangiogenesis‐related factors, GAPDH was used as the endogenous control, while for S1PRs, β‐actin was used as the endogenous control. The relative expression levels were calculated using the 2^−ΔΔCt^ method.

### 2.23 Statistical Analysis

All data were analyzed using GraphPad Prism 8.0. Normality was assessed with the Shapiro–Wilk test, and homogeneity of variances with the Brown–Forsythe test. For two‐group comparisons, an unpaired *t*‐test was used if both groups were normally distributed; otherwise, the Mann–Whitney test was applied. For comparisons of three or more groups, one‐way or two‐way ANOVA was employed when data were normally distributed and variances were equal. If variances were unequal, ANOVA with Brown–Forsythe and Welch corrections was used. Nonnormally distributed data were analyzed by the Kruskal–Wallis test followed by Dun's post hoc multiple comparisons. For in vivo experiments, the sample size was *n* ≥ 3 (representing the number of independent experimental animals), and for in vitro experiments, *n* ≥ 3 (representing the number of independent replicate experiments). Quantitative data are presented as mean ± SEM, and *p* < 0.05 was considered statistically significant.

## 3 Results

### 3.1 FTY720 Alleviates Autism‐Like Behaviors in MIA Offspring Mice

**FIGURE 1:**
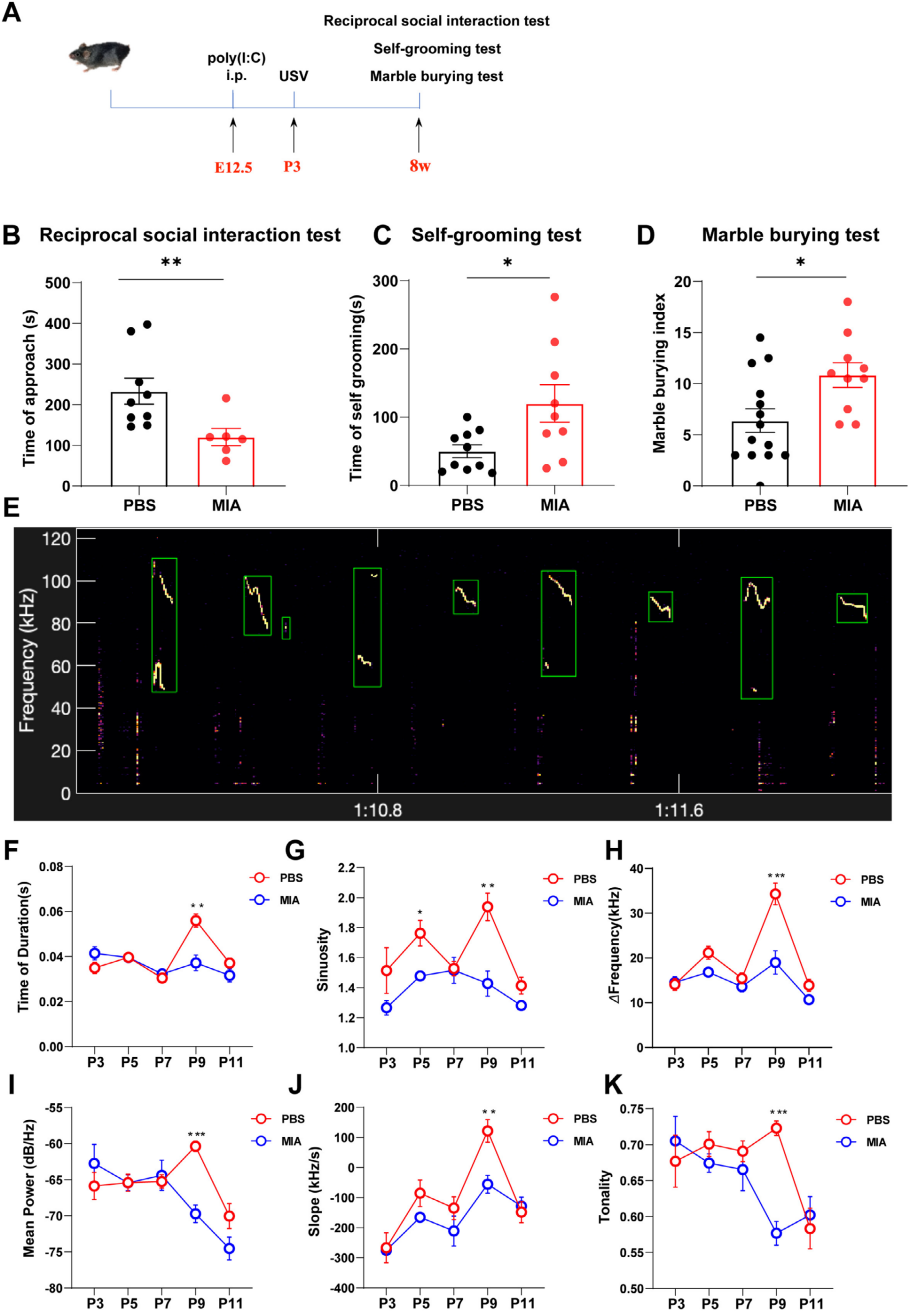
Behavioral validation of autism mice induced by maternal immune activation. (A) Flowcharts for the ultrasonic vocalization (USV) test, reciprocal social interaction test, self‐grooming test, and marble burying test. (B) Quantitative analysis of the time experimental mice spent closing to a novel conspecific in the reciprocal social interaction test. Each group consisted of 6–9 mice, and comparisons between groups were performed using the Kolmogorov–Smirnov test. **p < 0.01 compared to the PBS group. (C) Quantitative analysis of grooming time in the self‐grooming test. Each group consisted of 9–10 mice, and comparisons between groups were performed using Welch's *t*‐test. *p < 0.05 compared to the PBS group. (D) Quantitative analysis of the marble‐burying index in the marble‐burying test. Each group consisted of 10–14 mice, and comparisons between groups were performed using an Unpaired *t*‐test. *p < 0.05 compared to the PBS group. (E) Spectrogram of mouse ultrasonic vocalizations. (F–K) Quantitative statistical analysis of the duration (F), sinuosity (G), difference between high and low frequencies (H), mean power (I), slope (J), and tonality (K) in mice at postnatal Days 3, 5, 7, 9, and 11. Each group consisted of 10–13 mice, and comparisons between groups were performed using Mixed‐effects analysis. *p < 0.05, **p < 0.01, ***p < 0.001 compared to the PBS group.

Our previous study has demonstrated that MIA induces autism‐like behaviors in offspring mice through three‐chamber social interaction tests, self‐grooming tests, and marble burying assays [7]. The present research also showed that MIA‐induced offspring exhibited reduced social interaction time (social deficits; Figure 1B), prolonged grooming duration (repetitive stereotyped behaviors; Figure 1C), and increased marble burying frequency (anxiety‐like behaviors; Figure 1D); these assessments followed the experimental design illustrated in Figure 1A. To further confirm the successful establishment of an MIA‐induced offspring ASD model, we performed ultrasonic vocalization (USV) tests (Figure 1E). Statistical analysis revealed that on postnatal Day 9, MIA offspring mice exhibited significantly lower vocalization duration (Figure 1F), sinuosity (Figure 1G), frequency (Figure 1H), mean power (Figure 1I), slope (Figure 1J), and tonality (Figure 1K) compared to PBS offspring mice. These results confirm that MIA intervention successfully establishes an offspring mouse model of ASD.

**FIGURE 2:**
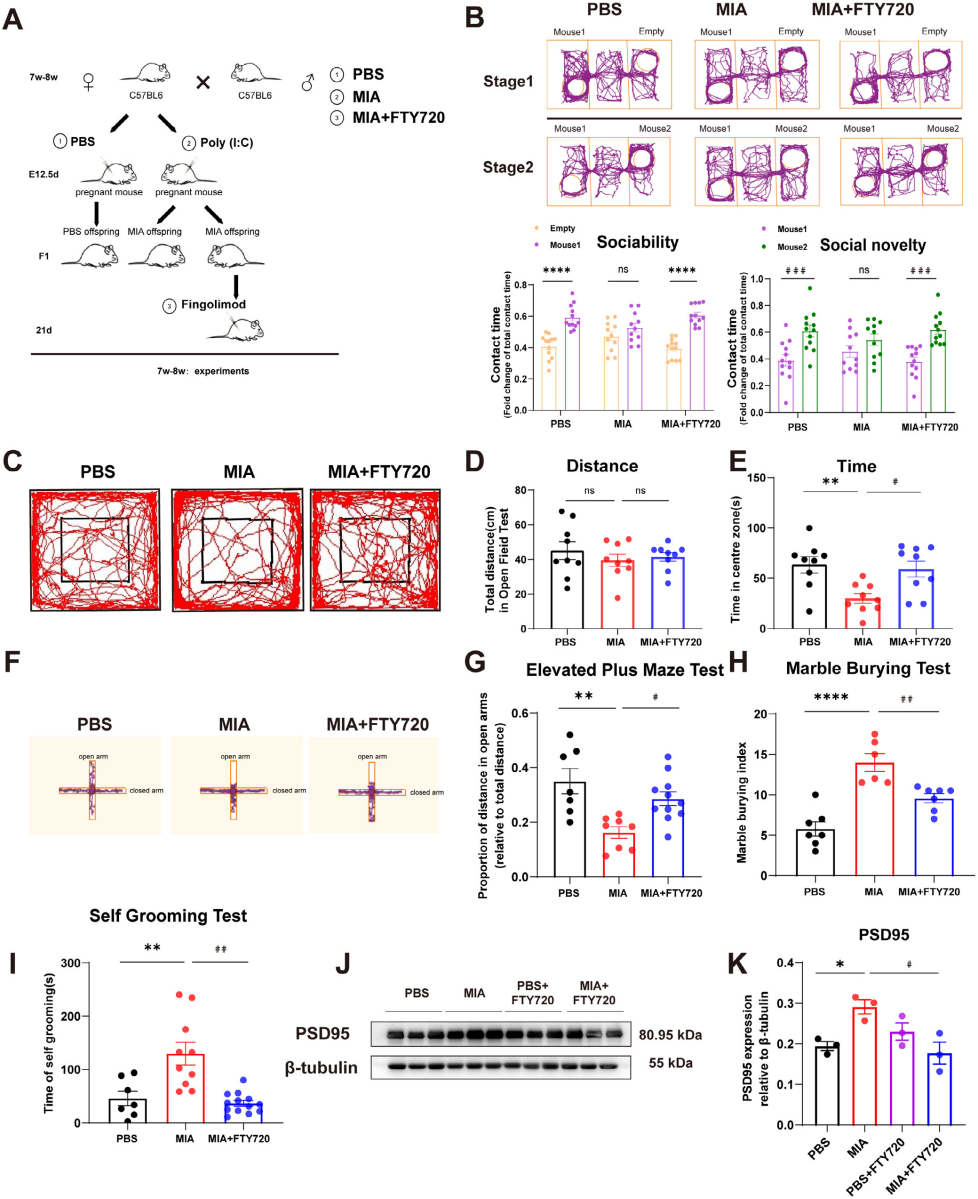
Effect of FTY720 on autism‐like pathological features in MIA offspring mice. (A) Flowchart for construction of experimental mouse models. (B) The ANY‐maze system recorded the mouse path in the three‐chamber social test; quantitative analysis of the time the experimental mouse spent in contact with the empty cage and the time spent in contact with Mouse 1 during stage 1. Each group consisted of 11–12 mice, and comparisons between groups were performed using Two‐way ANOVA followed by Sidak's multiple comparisons test for statistical analysis. ****p < 0.0001 compared to the Empty group. In stage 2, quantitative analysis was performed on the time the experimental mouse spent in contact with Mouse 1 and the time spent in contact with Mouse 2. Each group consisted of 11–12 mice, and comparisons between groups were performed using Two‐way ANOVA followed by Sidak's multiple comparisons test for statistical analysis. ^###^ *p* < 0.001 compared to the Mouse 1 group. (C) Images of the mouse trajectory in the open‐field test recorded by the TopScan Realtime Option Version 2.00 behavioral detection system. (D) Quantitative analysis of the total distance traveled by three groups of mice in the open‐field test. Each group consisted of 9 mice, and comparisons between groups were performed using One‐way ANOVA followed by Tukey's multiple comparisons test. (E) Quantitative analysis of the time spent in the central area of the open‐field test by three groups of experimental mice. Each group consisted of 9 mice, and comparisons between groups were performed using One‐way ANOVA followed by Tukey's multiple comparisons test. **p < 0.01 compared to the PBS group; ^#^ *p* < 0.05 compared to the MIA group. (F) Images of the mouse trajectory in the elevated plus maze test recorded by the Noldus EthoVision XT software system. (G) Quantitative analysis of the proportion of distance traveled in the open arms in the elevated plus maze test by three groups of experimental mice. Each group consisted of 7–11 mice, and comparisons between groups were performed using One‐way ANOVA followed by Tukey's multiple comparisons test. **p < 0.01 compared to the PBS group; ^#^ *p* < 0.05 compared to the MIA group. (H) Quantitative analysis of the marble‐burying index recorded in the marble‐burying test for three groups of experimental mice. Each group consisted of 6–7 mice, and comparisons between groups were performed using One‐way ANOVA followed by Tukey's multiple comparisons test. ****p < 0.0001 compared to the PBS group; ^##^ *p* < 0.01 compared to the MIA group. (I) Quantitative analysis of grooming time in the grooming test for three groups of experimental mice. Each group consisted of 7–13 mice, and comparisons between groups were performed using Brown–Forsythe and Welch ANOVA followed by Dunnett's multiple comparisons test. **p < 0.01 compared to the PBS group; ^##^ *p* < 0.01 compared to the MIA group. (J) Images of the WB experimental results for PSD95 content determination in the hippocampal region of four groups of experimental mice. (K) Quantitative analysis of PSD95 content in the hippocampal region of four groups of experimental mice. The data were obtained from three independent replicate experiments. Comparisons between groups were performed using Brown–Forsythe and Welch ANOVA followed by Dunnett's multiple comparisons test. *p < 0.05 compared to the PBS group; ^#^ *p* < 0.05 compared to the MIA group.

To investigate the therapeutic effects of FTY720 in MIA‐induced autistic offspring mice, postnatal Day 21 (P21) mice were intraperitoneally injected with FTY720, followed by behavioral assessment at 7–8 weeks using the three‐chamber social test (social interaction), open‐field test (exploratory behavior), elevated plus maze and marble burying tests (anxiety‐related states), and self‐grooming test (repetitive behaviors) (Figure 2A–I). Three‐chamber social tests showed that FTY720 significantly enhanced social interaction skills and social novelty preference in MIA offspring mice. Compared with untreated MIA offspring mice, FTY720‐treated MIA offspring mice spent significantly more time in the open‐field central zone and exhibited increased locomotor distance in the elevated plus maze. Moreover, FTY720 reduced marble burying indices and self‐grooming duration in MIA offspring mice. These results indicate that FTY720 ameliorates social cognitive deficits, exploratory impairments, and anxiety‐like/repetitive behaviors in MIA offspring mice. Clinical investigations have demonstrated abnormal synaptic function in ASD patients, with hippocampal synaptic protein PSD95 serving as a potential biomarker for autism [35]. The therapeutic efficacy of FTY720 in autism was further validated by quantifying hippocampal expression of synaptic protein PSD95 across offspring mouse groups (Figure 2J,K). The results showed a significant upregulation of PSD95 in the hippocampal tissue of MIA offspring mice, consistent with clinical observations. Notably, FTY720 treatment potently attenuated the MIA‐induced upregulation of hippocampal PSD95 protein in offspring mice. Collectively, these findings demonstrate the therapeutic efficacy of FTY720 against MIA‐induced ASD‐like phenotypes in mice.

### 3.2 FTY720 Ameliorates Ventricular Dilation in MIA Offspring Mice

**FIGURE 3:**
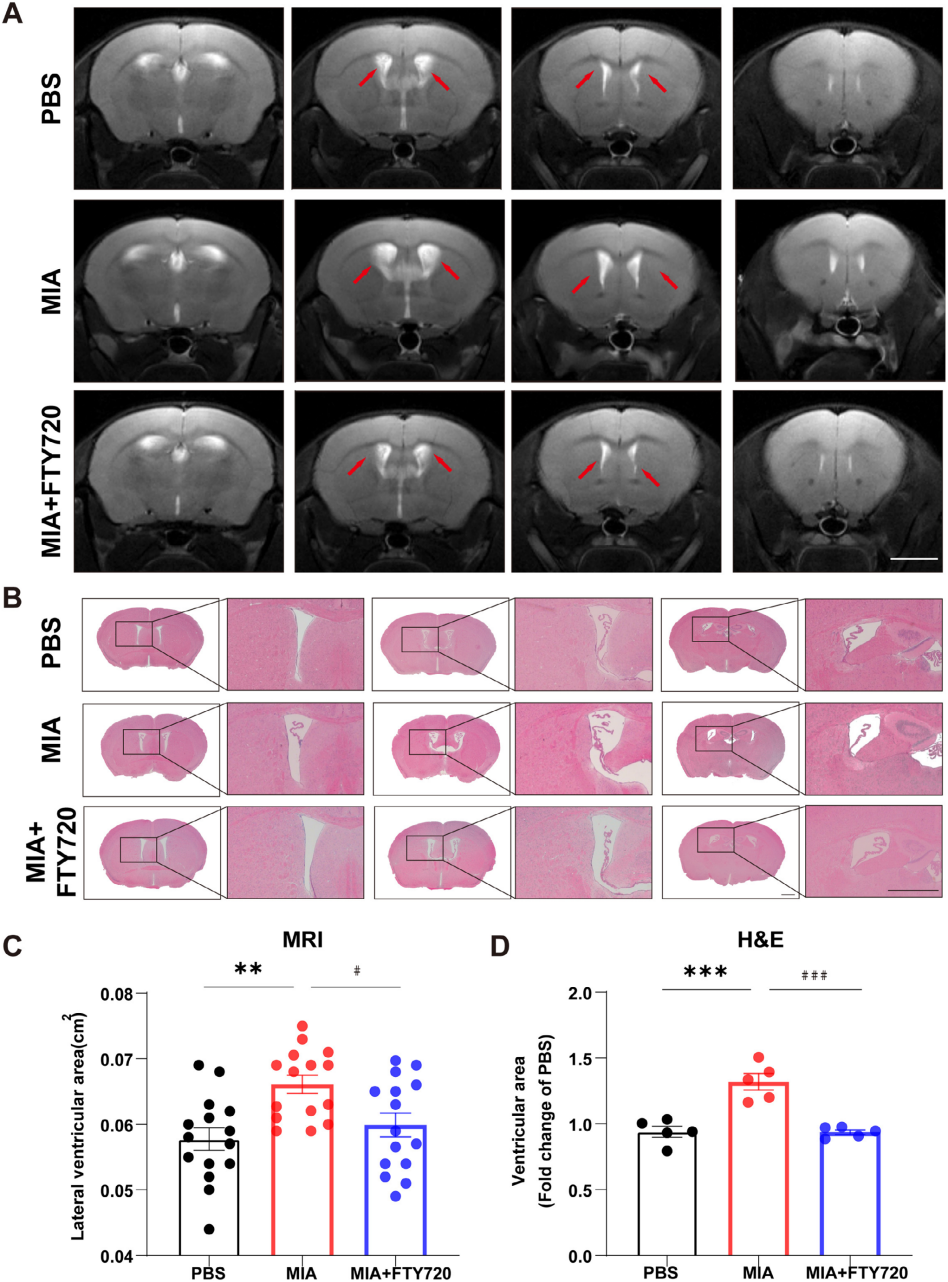
FTY720 ameliorates ventriculomegaly in MIA offspring mice. (A) Images of the lateral ventricles of three groups of experimental mice obtained by magnetic resonance imaging (MRI). Scale bar = 5 mm. (B) Images of the lateral ventricles of three groups of experimental mice obtained by H&E staining. Scale bar = 1 mm. (C) Quantitative analysis of the lateral ventricle area in three groups of experimental mice obtained by magnetic resonance imaging (MRI). Each group consisted of 15 mice, and comparisons between groups were performed using One‐way ANOVA followed by Tukey's multiple comparisons test. **p < 0.01 compared to the PBS group; ^#^ *p* < 0.05 compared to the MIA group. (D) Quantitative analysis of the lateral ventricle area in three groups of experimental mice obtained by H&E staining. Each group consisted of 5 mice, and comparisons between groups were performed using One‐way ANOVA followed by Tukey's multiple comparisons test. ***p < 0.001 compared to the PBS group; ^###^ *p* < 0.001 compared to the MIA group.

To investigate whether MIA offspring exhibit the pathological phenotype of ventriculomegaly, we employed a combination of 7T small‐animal magnetic resonance imaging (MRI) and histological assessment. MRI quantitative analysis revealed that the cross‐sectional area of the lateral ventricles was significantly larger in MIA offspring compared to the PBS control group, while the FTY720 treatment group showed a significant reduction compared to the MIA model group (Figure 3A,C). Histological confirmation via H&E staining further demonstrated that the lateral ventricle area was enlarged in the MIA model group compared to the control group, and this expansion was mitigated by FTY720 treatment (Figure 3B,D). Both imaging and histological evidence consistently indicate that FTY720 can effectively alleviate ventriculomegaly induced by MIA.

### 3.3 FTY720 Ameliorates Structural and Functional Impairments of Meningeal Lymphatics in MIA Offspring Mice

**FIGURE 4:**
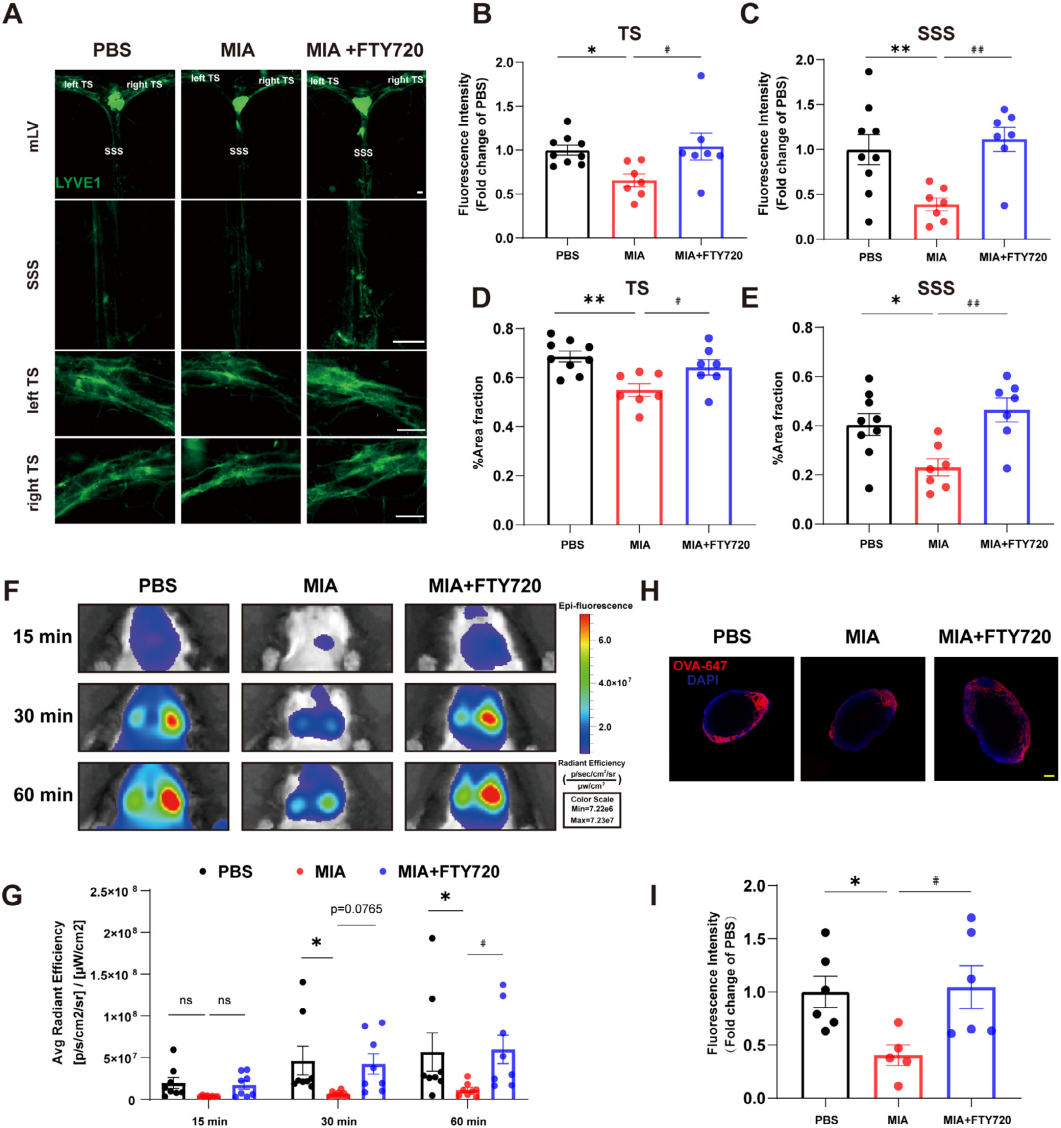
Effects of FTY720 on the structure and drainage function of meningeal lymphatic vessels in MIA offspring mice. (A) Images of LYVE1 immunofluorescence staining of the meningeal lymphatic vessels in three groups of experimental mice. Scale bar = 500 μm. (B, C) Quantitative analysis of LYVE1 fluorescence intensity in the superior sagittal sinus and transverse sinus of the meningeal lymphatic vessels in three groups of experimental mice. Data were obtained from 7 to 9 independent replicate experiments. Comparisons between groups were performed using One‐way ANOVA followed by Dunnett's multiple comparisons test. *p < 0.05, **p < 0.01 compared to the PBS group; ^#^ *p* < 0.05, ^##^ *p* < 0.05 compared to the MIA group. (D, E) Quantitative analysis of the percentage of LYVE1 fluorescent area in the transverse sinus and superior sagittal sinus of the meningeal lymphatic vessels in three groups of experimental mice. Data were obtained from 7 to 9 independent replicate experiments. Comparisons between groups were performed using One‐way ANOVA followed by Dunnett's multiple comparisons test. *p < 0.05, **p < 0.01 compared to the PBS group; ^#^ *p* < 0.05, ^##^ *p* < 0.01 compared to the MIA group. (F) Images of the fluorescence of the dye OVA‐647 in the deep cervical lymph nodes of three groups of experimental mice, imaged using a small‐animal in vivo visible light system at 15, 30, and 60 min after injection of the fluorescent dye OVA‐647 into the cisterna magna of the experimental mice. (G) Quantitative analysis of the content of the fluorescent dye OVA‐647 in the deep cervical lymph nodes of three groups of experimental mice at 15, 30, and 60 min after injection of the fluorescent dye OVA‐647. Data were obtained from eight independent replicate experiments. Comparisons between groups were performed using Two‐way ANOVA followed by Sidak's multiple comparisons test. *p < 0.05 compared to the PBS group; ^#^ *p* < 0.05 compared to the MIA group. (H) Images of the fluorescence of OVA‐647 dye in the neck region of three groups of experimental mice at 30 min after injection of the fluorescent dye OVA‐647. Scale bar = 100 μm. (I) Quantitative analysis of the fluorescence intensity of OVA‐647 dye in the deep cervical lymph nodes of three groups of experimental mice at 30 min after injection. Data were obtained from 5 to 6 independent replicate experiments. Comparisons between groups were performed using One‐way ANOVA followed by Dunnett's multiple comparisons test. *p < 0.05 compared to the PBS group; ^#^ *p* < 0.05 compared to the MIA group.

Research reports indicate that the accumulation of cerebrospinal fluid (CSF) due to CSF circulatory disorders is one of the critical etiological factors for ventricular dilation. However, meningeal lymphatic vessels, as crucial structures involved in cerebrospinal fluid (CSF) drainage and immune regulation, exert significant regulatory effects on CSF circulation and the brain's immune microenvironment. It is suggested that FTY720 may reduce maternal immune activation (MIA)‐induced ventricular dilation and ameliorate autism‐like behaviors in offspring mice by improving the structure and function of meningeal lymphatic vessels. LYVE1, a lymphatic vessel biomarker in meningeal tissue, was used for immunofluorescence staining to observe structural changes in meningeal lymphatic vessels before and after modeling and drug administration (Figure 4A–E). The result showed that compared with the PBS control group, the fluorescence intensity and fluorescence area percentage of LYVE1 in the transverse sinus and superior sagittal sinus of MIA offspring mice were significantly decreased. By contrast, in FTY720‐treated MIA offspring mice, LYVE1 fluorescence intensity and area percentage in these sinuses were significantly higher than those in the untreated MIA offspring group, indicating that FTY720 protects damaged meningeal lymphatic vessels in MIA offspring mice.

Subsequently, OVA‐647 (fluorescein 647‐labeled ovalbumin) was injected into the mouse cisterna medullaris, and in vivo visible light imaging was conducted at deep cervical lymph nodes at 15, 30 and 60 min post‐injection (Figure 4F,G). At 30 and 60 min, dye accumulation in deep cervical lymph nodes of MIA offspring mice was significantly lower than that in PBS control offspring mice, whereas FTY720‐treated MIA offspring showed markedly higher dye accumulation than untreated MIA mice. Additionally, deep cervical lymph nodes were harvested 30 min post‐injection for ex vivo fluorescence imaging, confirming that FTY720 significantly enhanced dye accumulation in MIA offspring lymph nodes (Figure 4H,I). These findings demonstrate that FTY720 potently improves meningeal lymphatic drainage function in MIA offspring mice.

### 3.4 FTY720 Promotes Lymphangiogenesis in INF‐γ‐Stimulated SVEC4‐10 Cells and Suppresses the Expression of Lymphangiogenesis‐Related Inhibitory Factors

**FIGURE 5:**
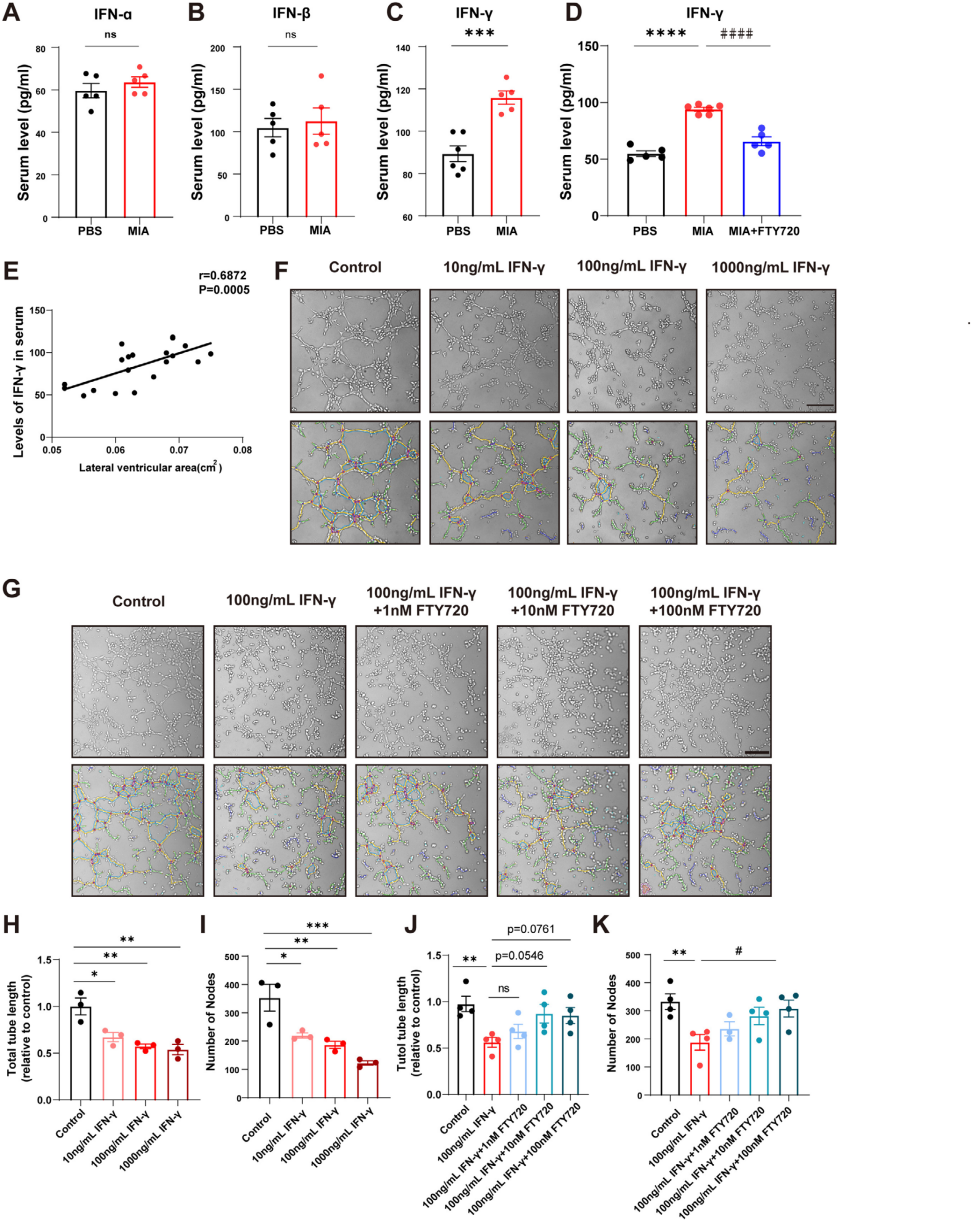
The in vitro model construction validated the effect of FTY720 in improving the tube formation ability of SVEC4‐10 cells stimulated by IFN‐γ. (A–C) Quantitative analysis of IFNs levels in the serum of PBS offspring mice and MIA offspring mice. Data were obtained from 5 to 6 independent replicate experiments. Comparisons between groups were performed using an Unpaired *t*‐test. ***p < 0.001 compared to the PBS group. (D) Quantitative analysis of IFN‐γ levels in the serum of three groups of mice. Data were obtained from 5 to 6 independent replicate experiments. Comparisons between groups were performed using One‐way ANOVA followed by Tukey's multiple comparisons test. ****p < 0.0001 compared to the PBS group; ^####^ *p* < 0.0001 compared to the MIA group. (E) Pearson correlation analysis between the lateral ventricle area and serum IFN‐γ levels in experimental mice, with a sample size of 20, *r* = 0.6872, *p* = 0.005. (F) Images of tube formation in SVEC4‐10 cells stimulated by gradient concentrations of IFN‐γ and images analyzed using the Angiogenesis Analyzer plugin for ImageJ. Scale bar = 500 μm. (G) Images of tube formation in SVEC4‐10 cells stimulated with 100 ng/mL IFN‐γ and treated with a concentration gradient of FTY720, images analyzed using the Angiogenesis Analyzer plugin for ImageJ. Scale bar = 500 μm. (H) Quantitative analysis of the total tube length in SVEC4‐10 cells stimulated by gradient concentrations of IFN‐γ. Data were obtained from three independent replicate experiments. Comparisons between groups were performed using One‐way ANOVA followed by Tukey's multiple comparisons test. *p < 0.05, **p < 0.01 compared to the Control group. (I) Quantitative analysis of the number of tube formation nodes in SVEC4‐10 cells stimulated by gradient concentrations of IFN‐γ. Data were obtained from three independent replicate experiments. Comparisons between groups were performed using One‐way ANOVA followed by Tukey's multiple comparisons test. *p < 0.05, **p < 0.01, ***p < 0.001 compared to the Control group. (J) Quantitative analysis of the total tube length in SVEC4‐10 cells stimulated with 100 ng/mL IFN‐γ and treated with a concentration gradient of FTY720. Data were obtained from four independent replicate experiments. Comparisons between groups were performed using One‐way ANOVA followed by Dunnett's multiple comparisons test. **p < 0.01 compared to the Control group. (K) Quantitative analysis of the number of tube formation nodes in SVEC4‐10 cells stimulated with 100 ng/mL IFN‐γ and treated with a concentration gradient of FTY720. Data were obtained from three to four independent replicate experiments. Comparisons between groups were performed using One‐way ANOVA followed by Tukey's multiple comparisons test. **p < 0.01 compared to the Control group; ^#^ *p* < 0.05 compared to the IFN‐γ group.

Our prior studies have demonstrated a significant enhancement of IFN‐γ signaling in the brain and serum of MIA offspring mice [7]. This study further confirmed elevated serum IFN‐γ levels in MIA offspring mice, with no significant changes observed in IFN‐α or IFN‐β. Notably, FTY720 treatment potently reduced serum IFN‐γ levels in MIA offspring mice (Figure 5A–D). Pearson correlation analysis further revealed a significant positive correlation between serum IFN‐γ levels and lateral ventricle area in these mice (Figure 5E). Collectively, these findings implicate IFN‐γ in the pathogenesis of MIA‐induced autism‐like phenotypes in offspring mice. To explore the regulatory effect of FTY720 on meningeal lymphatic vessels, we established an in vitro model by stimulating the lymphoendothelial cell line SVEC4‐10 (a standard model for lymphangiogenesis research [36]) with IFN‐γ. Tube formation assays showed that SVEC4‐10 cells treated with 100 and 1000 ng/mL IFN‐γ exhibited the most pronounced reduction in tubular structure length and branch node number (Figure 5F,H,I). A 100 ng/mL IFN‐γ concentration was selected for subsequent experiments to establish an isolated lymphatic injury model. Subsequent tube formation assays demonstrated that 100 nM FTY720 significantly increased tubular structure length and branch node number in IFN‐γ‐treated SVEC4‐10 cells (Figure 5G,J,K). These findings indicate that FTY720 enhances the tubulogenic capacity of lymphatic endothelial cells under IFN‐γ stress, suggesting a pro‐lymphangiogenic effect of FTY720 in SVEC4‐10 cells.

**FIGURE 6:**
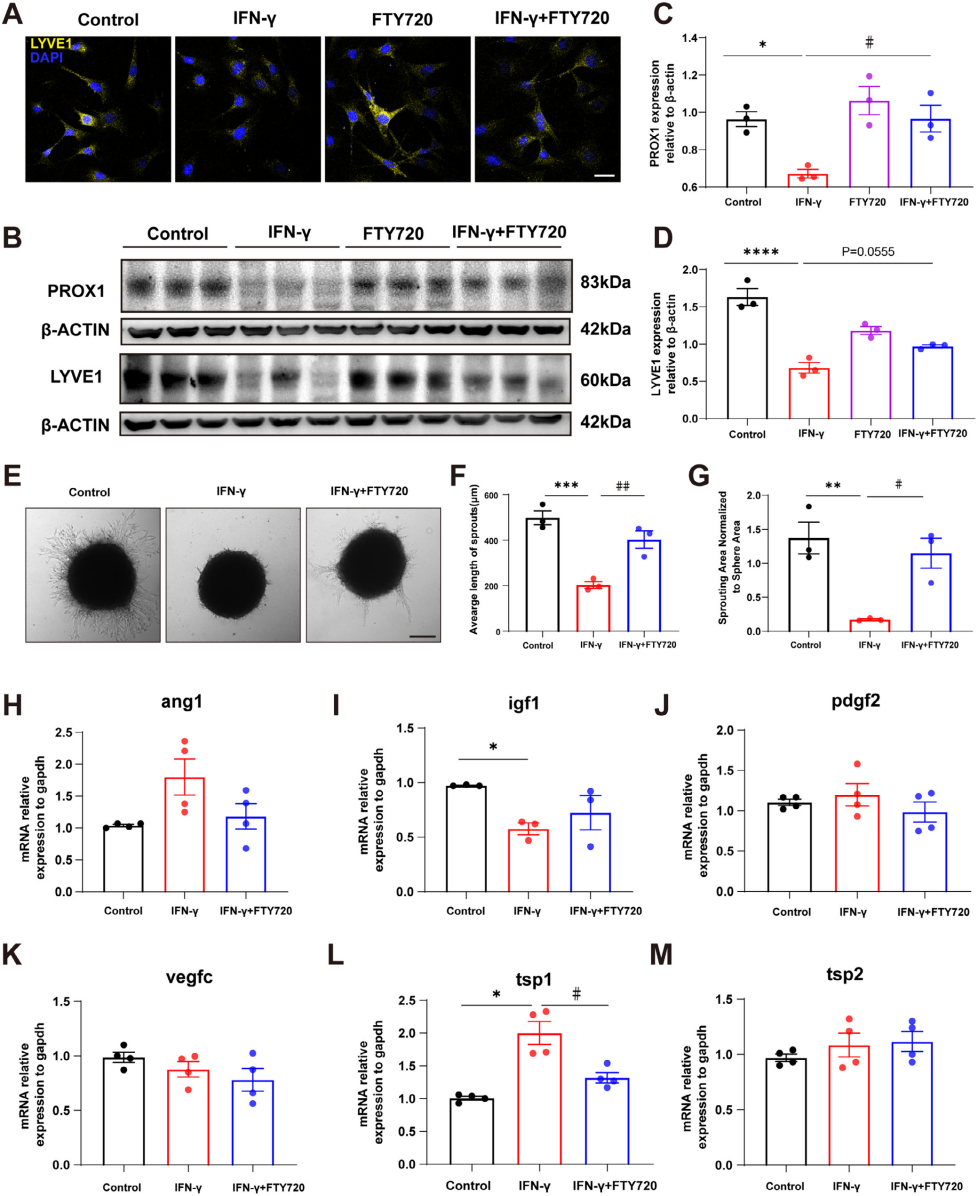
Ex vivo experiments confirm that FTY720 can ameliorate lymphangiogenic impairment in SVEC4‐10 cells stimulated by IFN‐γ. (A) Images of LYVE1 immunofluorescence in SVEC4‐10 cells subjected to four different treatments. Scale bar = 5 μm. (B) Images of WB experimental results for the content of lymphatic biomarkers PROX1 and LYVE1 in SVEC4‐10 cells subjected to four different treatments. (C, D) Quantitative analysis of the content of lymphatic biomarkers PROX1 and LYVE1 in SVEC4‐10 cells subjected to four different treatments. Data were obtained from three independent replicate experiments. Comparisons between groups were performed using One‐way ANOVA followed by Dunnett's multiple comparisons test. *p < 0.05, ****p < 0.0001 compared to the Control group; ^#^ *p* < 0.05 compared to the IFN‐γ group. (E) Images of sprouting in SVEC4‐10 cells subjected to three different treatments. Scale bar = 50 μm. (F) Quantitative analysis of the average sprouting length in SVEC4‐10 cells subjected to three different treatments. Data were obtained from three independent replicate experiments. Comparisons between groups were performed using One‐way ANOVA followed by Tukey's multiple comparisons test. ***p < 0.001 compared to the Control group; ^##^ *p* < 0.01 compared to the IFN‐γ group. (G) Quantitative analysis of the ratio of sprouting area to spheroid area in SVEC4‐10 cells subjected to three different treatments. Data were obtained from three independent replicate experiments. Comparisons between groups were performed using One‐way ANOVA followed by Tukey's multiple comparisons test. **p < 0.01 compared to the Control group; ^#^ *p* < 0.05 compared to the IFN‐γ group. (H–M) Quantitative analysis of mRNA levels of lymphangiogenesis‐related factors (ang1, angiopoietin‐1; igf1, insulin‐like growth factor‐1; pdgf2, platelet‐derived growth factor‐2; tsp1, thrombospondin‐1; tsp2, thrombospondin‐2; vegfc, vascular endothelial growth factor C) in SVEC4‐10 cells subjected to three different treatments. Data were obtained from 3 to 4 independent replicate experiments. Comparisons between groups were performed using One‐way ANOVA or Brown–Forsythe and Welch ANOVA followed by Dunnett's multiple comparisons test. *p < 0.05 compared to the Control group; ^#^ *p* < 0.05 compared to the IFN‐γ group.

LYVE‐1 and PROX1 are proteins vital for lymphatic vessel development and maintenance [23, 24]. Immunofluorescence and Western blot analyses revealed that FTY720 reversed IFN‐γ‐mediated downregulation of LYVE‐1 and PROX1 protein expression in SVEC4‐10 cells (Figure 6A–D). Sphere sprouting assays further showed that IFN‐γ stimulation significantly reduced sprout length and the ratio of sprout area to sphere area, whereas FTY720 potently inhibited IFN‐γ‐mediated decreases in both sprout length and the sprout area‐to‐sphere area ratio (Figure 6E–G). Collectively, these results demonstrate that FTY720 promotes lymphangiogenesis in IFN‐γ‐stimulated SVEC4‐10 cells.

To investigate the mechanism by which FTY720 promotes lymphangiogenesis in IFN‐γ‐stimulated SVEC4‐10 cells, we measured the expression of pro‐lymphangiogenic factors (ang1, igf1, pdgf2, and vegfc) and lymphangiogenesis‐related inhibitory factors (tsp1 and tsp2). Results showed that IFN‐γ treatment significantly upregulated the mRNA expression of lymphangiogenesis inhibitor TSP1 and downregulated pro‐lymphangiogenic factor IGF1. Notably, FTY720 reversed only the IFN‐γ‐mediated upregulation of TSP1 mRNA expression (Figure 6H–M). These findings suggest that FTY720 promotes lymphangiogenesis in IFN‐γ‐stimulated SVEC4‐10 cells possibly by inhibiting the expression of lymphangiogenesis‐related inhibitory factors.

### 3.5 FTY720 Promotes Lymphangiogenesis in INF‐γ‐Stimulated SVEC4‐10 Cells Targeting on S1PR3

**FIGURE 7:**
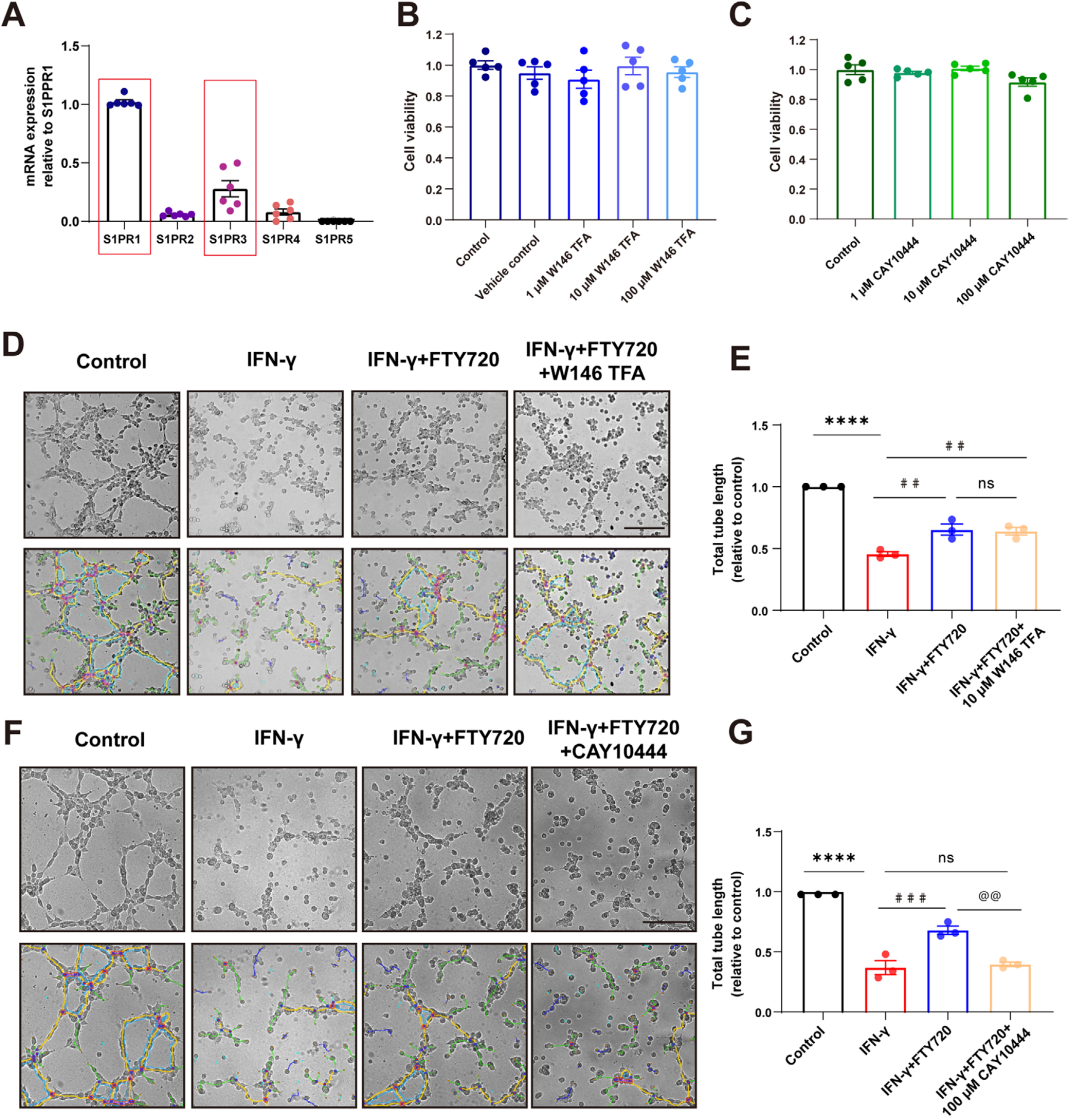
Impact of blocking S1PR1 or S1PR3 on the ameliorative effects of FTY720 on lymphangiogenic damage in SVEC4‐10 cells stimulated by IFN‐γ. (A) Quantitative analysis of the mRNA expression levels of S1PRs in the meninges of normal mice relative to the mRNA expression level of S1PR1. Data were obtained from six independent replicate experiments. (B, C) The cell viability of SVEC4‐10 cells under different treatment conditions was detected using the CCK8 assay, and the viability was quantitatively analyzed. Data were obtained from five independent replicate experiments. Comparisons between groups were performed using One‐way ANOVA followed by Tukey's multiple comparisons test. (D) Images of tube formation in SVEC4‐10 cells subjected to four different treatments and images analyzed using the Angiogenesis Analyzer plugin for ImageJ. Scale bar = 500 μm. (E) Quantitative analysis of the total tube length in SVEC4‐10 cells subjected to four different treatments. Data were obtained from three independent replicate experiments. Comparisons between groups were performed using One‐way ANOVA followed by Tukey's multiple comparisons test. ****p < 0.0001 compared to the Control group; ^##^ *p* < 0.01 compared to the IFN‐γ group. (F) Images of tube formation in SVEC4‐10 cells subjected to four different treatments and images analyzed using the Angiogenesis Analyzer plugin for ImageJ. Scale bar = 500 μm. (G) Quantitative analysis of total tube length in SVEC4‐10 cells subjected to four different treatments. Data were obtained from three independent replicate experiments. Comparisons between groups were performed using One‐way ANOVA followed by Tukey's multiple comparisons test. ****p < 0.0001 compared to the Control group; ^###^ *p* < 0.001 compared to the IFN‐γ group; ^@@^ *p* < 0.01 compared to the IFN‐γ + FTY720 group.

To identify the target of FTY720 in regulating lymphangiogenesis, RNA was extracted from the meninges of normal mice to measure the expression levels of five classic S1PRs (Figure 7A). Results showed that S1PR1 had the highest mRNA expression in mouse meninges, followed by S1PR3. These findings were consistent with endothelial cell S1PR expression data from the Brain RNA‐Seq database. Subsequently, we treated SVEC4‐10 cells with S1PR1 antagonist W146 TFA and S1PR3 antagonist CAY10444 respectively to observe whether the regulatory effect of FTY720 on the tubule‐forming ability of SVEC4‐10 cells after IFN‐γ stimulation still existed. First, cytotoxicity assays confirmed that three concentrations of W146 TFA and CAY10444 had no toxic effects on SVEC4‐10 cells (Figure 7B,C). Tube formation assays showed that W146 TFA did not affect FTY720‐promoted tubulogenesis in IFN‐γ‐stimulated cells (Figure 7D,E), whereas CAY10444 blocked this effect (Figure 7F,G). Collectively, these results demonstrate that FTY720 promotes lymphangiogenesis in IFN‐γ‐stimulated SVEC4‐10 cells via S1PR3.

## 4 Discussion

The MIA offspring autism model is established by administering immune activators (e.g., LPS or Poly I:C) to pregnant mice, inducing maternal systemic immune inflammation that disrupts fetal nervous system development and leads to ASD‐like pathological and behavioral phenotypes in offspring [37, 38]. FTY720, the first orally approved medication for treating Multiple Sclerosis (MS) [19]. By targeting Sphingosine‐1‐Phosphate Receptors (S1PRs) on immune cell surfaces, FTY720 inhibits immune cell infiltration into the central nervous system and thereby exerting immunosuppressive and anti‐inflammatory regulatory effects [39]. Increasing evidence shows that FTY720 has shown promise in treating other immunomodulatory diseases [19, 40, 41]. Our research confirms that FTY720 significantly ameliorates autism‐like behaviors in MIA offspring mice, consistent with prior reports [23]. However, the underlying therapeutic mechanisms of FTY720 in this model remain unelucidated and require further investigation.

Clinical studies have shown that patients with ASD exhibit neuroimaging features of ventricular enlargement, and a significant correlation has been identified between ventricular dilation in children with ASD and various behavioral deficits [3, 42]. Our findings also demonstrate ventricular enlargement in MIA‐induced autistic offspring mice, consistent with the initial observations first reported by our laboratory [7]. However, whether this feature is universal across other types of ASD models remains to be validated, and the causal relationship between ASD and ventricular dilation urgently requires in‐depth exploration.

Literature reports indicate that structural abnormalities of the ventricular wall caused by neurodevelopmental disorders disrupt normal CSF circulation by affecting CSF flow dynamics [43]. Ventricular dilation resulting from abnormal CSF circulation can impair neurogenesis or neural regeneration in the Subventricular Zone (SVZ), thereby further affecting neurological function. Our previous studies have confirmed that MIA offspring mice exhibit structural and functional abnormalities in the choroid plexus and ependyma [7], which represent critical causes of ventricular enlargement. Meningeal lymphatic vessels serve as key structures for fluid exchange, metabolic waste clearance, and immune cell trafficking in the central nervous system. They drain metabolic waste and immune cells from CSF to Deep Cervical Lymph Nodes (dCLN), thereby regulating central nervous system homeostasis [44, 45]. Existing studies have shown that meningeal lymphatic vessels clear Aβ protein and tau protein from brain interstitial fluid via CSF drainage, maintaining a balanced neurofunctional environment and supporting brain health in Alzheimer's disease patients [46]; dysfunction of meningeal lymphatic drainage exacerbates α‐syn pathology, promoting the progression of Parkinson's disease [14]; and following subarachnoid hemorrhage, meningeal lymphatic vessels drain extravasated red blood cells from CSF into dCLN, alleviating the severity of subarachnoid hemorrhage [47]. However, despite being an important pathway for CSF drainage, the role of meningeal lymphatic vessels in the pathophysiology of ASD remains unreported in the literature. Our study for the first time confirms structural and functional impairments in meningeal lymphatic vessels of MIA offspring mice, and demonstrates that FTY720 preserves the structural integrity of meningeal lymphatic vessels and improves their drainage function. These results indicate that FTY720's regulation of meningeal lymphatic vessel structure and function represents a key mechanism for ameliorating autism‐like behaviors and ventricular dilation in MIA offspring mice. These findings not only reveal dysfunction of the meningeal lymphatic system as an important underlying pathological mechanism for ventricular enlargement in ASD but also provide a new theoretical basis for targeted ASD therapy. However, whether this discovery is universal across other ASD model animals or patients remains to be verified. Future studies may focus on investigating the occurrence of ventricular enlargement in different types of ASD models, the neuroimaging characteristics of meningeal lymphatic vessels in ASD patients, and the relationship between lymphatic system dysfunction and meningeal lymphatic vessel injury.

Our previous RNA‐seq analysis of choroid plexus from adult offspring of PBS and MIA mice revealed significant enrichment of antiviral and IFN signaling pathways, and both qPCR and serum assays confirmed the most pronounced change in IFN‐γ; furthermore, blocking the IFN‐γ receptor markedly alleviated autism‐like behaviors and ventricular enlargement in MIA offspring, indicating that IFN‐γ signaling plays a critical role in the pathological process [7]. Therefore, to further investigate the regulatory mechanism of FTY720 on the structure and function of meningeal lymphatic vessels, we established an in vitro model by stimulating lymphatic endothelial cells (SVEC4‐10) with IFN‐γ. The results demonstrated that FTY720 could reverse the inhibitory effect of IFN‐γ on tube formation and sprouting in SVEC4‐10 cells. Moreover, FTY720 was found to suppress the IFN‐γ‐mediated upregulation of *tsp1*, a lymphangiogenesis‐related inhibitor, in SVEC4‐10 cells. These findings indicate that FTY720 promotes lymphatic endothelial tube formation by inhibiting the expression of lymphangiogenesis‐related inhibitors, thereby maintaining the structural integrity of lymphatic vessels and improving their drainage function. The effects of FTY720 could be blocked by CAY10444, suggesting that FTY720 exerts its regulatory effect on lymphatic vessels by directly acting on the S1PR3 receptor of lymphatic endothelial cells.

However, FTY720 is a “pan” S1P‐receptor modulator with high affinity for S1PR1/3/4/5 and is rapidly converted to FTY720‐P in vivo [48, 49, 50], thereby broadly suppressing the trafficking and function of T cells [51], B cells [52], dendritic cells [53] and myeloid cells [54]. Autism spectrum disorder is increasingly regarded as a chronic, low‐grade inflammatory condition, and the meningeal lymphatic lumen itself contains neutrophils [55], T cells [13] and antigen‐presenting cells [56]. Thus, the immunosuppressive action of FTY720 on these immune populations could, in principle, contribute to its therapeutic effect in ASD, although we did not experimentally verify this possibility in the present study. Unfortunately, this same polypharmacology underlies many of its clinical adverse effects: recent pharmaco‐epidemiologic surveys have linked FTY720 therapy to increased risks of outpatient infections, cerebrovascular events, ischemic heart disease and hypertension [57, 58]. Building on our current findings, future work should therefore develop either pure S1PR3‐selective agonists targeting meningeal‐lymphatic endothelial cells or engineer FTY720‐derived S1PR3‐specific pro‐drugs, enabling lymphatic‐restricted delivery and minimizing the liability cascade that arises from simultaneous engagement of multiple S1P receptors.

## 5 Conclusion

This study demonstrates that FTY720 ameliorates autism‐like behaviors, ventricular dilation, and meningeal lymphatic vessel injury in MIA offspring mice. It further reveals that FTY720 promotes lymphangiogenesis by targeting S1PR3 to downregulate tsp1 (a lymphangiogenesis inhibitor), thereby reversing lymphatic vessel impairment.

## Author Contributions

Chen Hong: conceptualization, data curation, methodology, validation, formal analysis, writing – review and editing. Han‐Lian Xiao: methodology, validation, data curation. Zhi‐Hui Sun: validation, writing – review and editing. Ruo‐Bing Guo: validation, writing – review and editing. Xi‐Yue Zhang: conceptualization, writing – review and editing, methodology. Juan Ji: writing – review and editing, methodology. Xiu‐Lan Sun: conceptualization, validation, resources, writing – review and editing, supervision, project administration, funding acquisition.

## Ethics Statement

All animal experimental protocols were approved by the review committee of Nanjing Medical University (IACUC2004037‐1) and complied with institutional guidelines.

## Conflicts of Interest

The authors declare no conflicts of interest.

## Data Availability

The data that support the findings of this study are available from the corresponding author upon reasonable request.
